# Single-Domain Antibody Multimers for Detection of Botulinum Neurotoxin Serotypes C, D, and Their Mosaics in Endopep-MS

**DOI:** 10.3390/toxins15090573

**Published:** 2023-09-17

**Authors:** Michiel M. Harmsen, Jan C. Cornelissen, Fimme J. van der Wal, Jan H. W. Bergervoet, Miriam Koene

**Affiliations:** 1 Wageningen Bioveterinary Research, Wageningen University & Research, 8221 RA Lelystad, The Netherlandsfimme.vanderwal@wur.nl (F.J.v.d.W.); 2 Wageningen Plant Research, Wageningen University & Research, 6708 PB Wageningen, The Netherlands

**Keywords:** botulinum neurotoxin group III, mosaic toxin, botulism, *Clostridium*, single-domain antibody, nanobody, VHH, multimerization

## Abstract

Botulinum neurotoxins (BoNTs) are highly toxic proteins that require high-affinity immunocapture reagents for use in endopeptidase-based assays. Here, 30 novel and 2 earlier published llama single-domain antibodies (VHHs) against the veterinary-relevant BoNT serotypes C and D were yeast-produced. These VHHs recognized 10 independent antigenic sites, and many cross-reacted with the BoNT/DC and CD mosaic variants. As VHHs are highly suitable for genetically linking to increase antigen-binding affinity, 52 VHH multimers were produced and their affinity for BoNT/C, D, DC, and CD was determined. A selection of 15 multimers with high affinity (*K_D_* < 0.1 nM) was further shown to be resilient to a high salt wash that is used for samples from complex matrices and bound native BoNTs from culture supernatants as shown by Endopep-MS. High-affinity multimers suitable for further development of a highly sensitive Endopep-MS assay include four multimers that bind both BoNT/D and CD with *K_D_* of 14–99 pM, one multimer for BoNT/DC (65 pM) that also binds BoNT/C (75 pM), and seven multimers for BoNT/C (<1–19 pM), six of which also bind BoNT/DC with lower affinity (93–508 pM). In addition to application in diagnostic tests, these VHHs could be used for the development of novel therapeutics for animals or humans.

## 1. Introduction

Botulinum neurotoxins (BoNTs) are produced by anaerobic spore-forming Clostridia, including *Clostridium botulinum*, *C. butyricum*, and *C. baratii*. The ingestion of food or feed contaminated with BoNTs can cause flaccid paralysis, called botulism, which can lead to death by respiratory failure [1,2]. BoNTs are among the most potent bacterial toxins known [3]. BoNTs are traditionally grouped into seven serotypes, A−G, based on recognition by antisera. The toxins of different serotypes are zinc metalloproteases that possess differential protease specificity; BoNT/A, C, and E cleave synaptosomal-associated protein (SNAP)-25, and BoNT/B, D, F, and G cleave synaptobrevin-2 (also known as VAMP-2). In addition to SNAP-25, BoNT/C also cleaves syntaxin [4].

The structure of BoNTs was recently reviewed [1,2]. The 150-kDa BoNT holotoxin consists of a 50-kDa light chain (LC) and a 100-kDa heavy chain (HC). The LC and HC are linked by a disulfide bond and are complexed with accessory proteins that allow BoNT to persist in the environment. The HC is composed of a 50-kDa N-terminal domain (H_N_) and a 50-kDa C-terminal domain (H_C_). The various domains have specific functions. The H_C_ domain enables binding to gangliosides on neurons. The H_N_ domain is responsible for the translocation of the LC into the cytoplasm. Finally, the LC proteolyzes the intracellular synaptic vesicle protein SNAP-25 or synaptobrevin-2 resulting in the inhibition of neurotransmitter release into the synaptic cleft causing flaccid paralysis. Many other bacterial toxins, including the diphtheria toxin, anthrax lethal and edema toxins, *C. difficile* toxin A and tetanus neurotoxin (TeNT), are similarly composed of three functional domains [5,6].

BoNT/A, B, E, F and G are mainly associated with botulism in humans whereas BoNT/C, D, CD and DC are associated with animal botulism. BoNT/DC and CD are naturally existing mosaic toxins related to BoNT/C and D [7]. The BoNT/DC LC and H_N_ domains are almost identical to BoNT/D, whereas its H_C_ domain (H_C_/DC) shares about 77% amino acid sequence identity with H_C_/C. The protein sequences of BoNT/CD are almost identical to LC/C, H_N_/C and H_C_/D. *C. botulinum* strains can be classified into different groups based on various characteristics. The so-called group III strains produce BoNT/C and D and their mosaic variants [8,9]. The work presented here focuses on diagnostics for group III BoNTs.

Due to their high toxicity, there is a need for highly sensitive diagnostics for BoNT detection and identification. For laboratory detection of BoNTs the mouse bioassay is still the gold standard, despite international efforts to develop alternative in vitro tests to reduce animal testing. Although many alternative technologies have been developed, reliable detection of all (sub)serotypes in complex clinical and environmental matrices remains a challenge. One of the most promising methods is the mass spectrometry (MS)-based endopeptidase BoNT activity assay known as Endopep-MS [10]. Endopep-MS detects the enzymatic action of the LC on a peptide substrate which mimics BoNTs in vivo protein target. By using BoNT-specific peptide substrates and monitoring the cleavage position by examining the mass of the N-terminal (NT) and C-terminal (CT) cleavage products, the various BoNTs can be differentiated. An immunoaffinity step, with capture antibodies on magnetic beads, prior to incubation with the peptide substrate, is a crucial aspect for detecting and differentiating BoNTs in clinical specimens and culture supernatants [11,12,13]. The immunoaffinity not only increases the sensitivity of the test by concentrating BoNT, but it also allows a washing step to remove non-specific proteases that are often present in clinical samples. The sensitivity of Endopep-MS has been shown to equal or exceed that of the mouse bioassay [13,14,15,16,17,18,19], has shown good performance in an international proficiency test for detection of BoNT/A, B and E [20], and compares favorably to other in vitro methods for routine diagnostics of clinical specimens and food [21,22].

While most advances have been made to improve the Endopep-MS assay for BoNT types related to human botulism [12,14,23], detection and identification of group III BoNTs related to animal botulism has also progressed [13,14,16]. A major challenge is the interference of non-specific proteases that are present in many complex sample matrices, like gastrointestinal contents, liver, feed and environmental samples [24]. These proteases not only may degrade the capture antibodies on the magnetic beads but can degrade the peptides used as a substrate for the enzymatic cleavage by BoNT. A wash with 2 M NaCl in PBS (high salt wash) after immunocapture of BoNTs from complex matrices has been shown to reduce non-specific protease activity [13,16,25]. Endopep-MS was also improved by the optimization of peptide substrates [19,26]. For efficient immunocapture, especially for BoNT/A, B, E and F, monoclonal antibodies (mAbs) have been optimized by increasing affinity using various molecular evolution approaches. Large increases in affinity have been obtained, resulting in up to 1000-fold lower affinity constants (*K_D_*) that are due to both an increase in association (*k_a_*) and a decrease in dissociation (*k_d_*) rate constant [11,27,28]. Also, for group III BoNTs capture mAbs have been developed that perform well in Endopep-MS [14,15,16,26]. More recently, mAbs against group III BoNTs were affinity matured for human therapeutic application [29]. However, the utilization of many of the published mAbs is conditional, which makes them less suitable for use in routine diagnostics.

In addition to conventional mAbs, various recombinant antibodies against BoNT have been isolated for use in diagnostics or therapy. This includes single-domain antibodies (VHHs) that are derived from camelid heavy-chain antibodies. These VHHs are mostly directed against BoNT/A, B, E and F, whereas some bind group III BoNTs [30,31,32,33,34,35,36]. Due to their single-gene origin, VHHs are highly suitable for the genetic fusion of different VHHs into multimers to increase affinity for their targets up to 1000-fold [37,38,39,40]. Multimerization has also been successfully applied to increase the neutralization of several bacterial toxins in animal disease models, including anthrax [41], ricin [42,43], Shiga toxin [44], TeNT [5], *C. difficile* toxin A [45] and BoNT/A [46].

To enable development and, ultimately, implement in-house routine diagnostics for BoNTs, novel antibodies were generated. In this work, 34 VHHs were isolated that bind group III BoNTs (30 VHHs), BoNT/A (3 VHHs) or BoNT/B (1 VHH). These 34 VHHs and 2 published VHHs [30] were yeast-produced and their antigenic specificity was characterized. VHH binding independent antigenic sites were used for generating 52 VHH multimers by genetic fusion of 2 or 3 VHHs. A selection of 15 multimers with high affinity (*K_D_* < 0.1 nM) for various group III BoNTs was further evaluated for use in routine diagnosis by Endopep-MS.

## 2. Results

### 2.1. Isolation of Monomeric VHHs

Novel VHHs binding to BoNTs were obtained by llama immunization with recombinant BoNT proteins that contained three mutations in the light chain for toxin inactivation. These inactivated recombinant BoNTs are referred to by the suffix i. Phage display selections from immune libraries, using holotoxin as well as its H_C_ domain, resulted in 91 unique VHHs, encompassing 6 VHHs against BoNT/Ai, 6 VHHs against BoNT/Bi, 40 VHHs against BoNT/Ci and 39 VHHs against BoNT/Di. Unique VHHs were given a name starting with the letter B for BoNT, then a letter to indicate the BoNT serotype used for their selection and subsequently a unique number. In total, 34 novel VHH clones were selected for yeast expression based on their BoNT binding in ELISA (results not shown) and sequence diversity (Table 1). The VHHs BC24F and BC22F, based on the sequences of the VHHs C24 and C22 binding to BoNT/C and published earlier [30], were also selected for yeast expression. The 34 novel VHHs and 2 VHHs published earlier formed 28 complementarity determining region 3 (CDR3) groups, A to AB. Clones from the same CDR3 group had at least 7 amino acid differences in the complete VHH sequence. Such clones always originate from the same llama (Table 1) consistent with the notion that they originate from the same B-cell clone. The order of VHHs in Table 1 is based on their BoNT specificity and grouping according to CDR3 and antigenic site (see below). This order was retained in all figures and tables.

### 2.2. Characterization of Yeast-Produced VHHs

VHHs were produced in yeast as a fusion to the long hinge region containing a single cysteine, enabling a partial dimerization [47] which is suitable for a direct VHH coating [48]. Only BC24F contains a potential N-glycosylation site (at IMGT position 60), which potentially compromises the antigen binding [49,50]. This VHH was found to be partially (about 50%) N-glycosylated with high mannose outer chains since endoglycosidase H treatment of BC24F resulted in disappearing of the band above the 97-kDa marker representing highly glycosylated VHH and a concomitant higher intensity of the band at about 24 kDa representing unglycosylated VHH (Figure S1A) as compared to untreated BC24F. BD188F migrated at about 66 kDa, indicating it is not a monomeric VHH (Figure S1C). All other VHHs migrated at about 24 kDa as a broad band (Figure S1A–C) representing monomeric VHHs with heterogeneous O-glycosylation in the hinge region [47].

**Table 1:** Phage display selection and sequence information of 34 novel VHHs and 2 VHHs published earlier.

VHH ^a^	Llama	Phage Display Selection ^b^	CDR3 Sequence	CDR3 Group	Different Residues in CDR3 Group ^c^	VHH Sub-Family ^d^
BoNT/A						
BA104	9245	BoNT/Ai	QSSGQPAT	A	n.a. ^e^	C
BA103	9245	BoNT/Ai	DAEVHKTSSDHALGIVYDY	B	n.a.	2
BA101	9245	BoNT/Ai	ATDMVHMRCDTYYSGSYYREKNDY	C	n.a.	3
BoNT/B						
BB109	9245	BoNT/Bi	SMKKAITDSAY	D	n.a.	2
BoNT/C						
BC141	9246	BC24F-H_C_/C	AASIHRAVGAYYSGSYYYDY	O	n.a.	1
BC126	9246	BC24F-BoNT/Ci	AAALIGDPRYGTRWYEYDY	Q	n.a.	1
BC147	9246	BC24F-H_C_/C	NKVNARAYDY	N	n.a.	2
BC146	9246	BC24F-H_C_/C	NADLPAGWGTLKTIDY	I	n.a.	2
BC112	9246	BC24F-H_C_/C	ASRRGWSFSTVTGGGSYDY	J	n.a.	1
BC118	9246	BC24F-H_C_/C	AAERDPSCGSSWYGGLRYDY	K	11	3
BC122	9246	BC24F-H_C_/C	AAERDPSCGSSWYGGLRYDY	K	11	3
BC123	9246	BC24F-H_C_/C	AAERDPTCWDDSGTSYYYGMRY	M	n.a.	3
BC143	9245	BoNT/Ci	NKAPVPWP	G	9	2
BC124	9245	BoNT/Ci	NKAPLPWP	G	9	X
BC125	9246	BoNT/Ci	AARLLRSGAYTYSDIQNYDY	H	10	1
BC145	9246	BoNT/Ci	AARLLRSGAYTYSDIQNYDY	H	10	1
BC134	9246	BoNT/Ci	AADSYYPLTSTPRY	L	20	1
BC139	9246	BoNT/Ci	AADPYYPLPTAARY	L	20	1
BC119	9245	BoNT/Ci	AKFKDRGFYRNTPGSSNYDN	P	n.a.	C
BC24	-	BoNT/C	AARRMDSGSYRYNDRETYDY	E	n.a.	X
BC22	-	BoNT/C	AVLREGGIYSSSSYAY	F	n.a.	X
BoNT/D						
BD156	9245	BoNT/Di	AAARYATCWRWSSRSYDL	R	7	3
BD165	9245	BoNT/Di	AAARYATCWRWSSRSYDY	R	7	3
BD157	9246	BoNT/Di	AAEPKYRAFFRSREEIFASMDY	U	n.a.	3
BD166	9246	BoNT/Di	AAITSWTEDAANCQGFDY	S	n.a.	3
BD155	9246	BoNT/Di	AAPCNYPVS	T	n.a.	3
BD186	9246	BD157F-BoNT/Di	AASAPHTIVVDSPWDY	Z	16	1
BD188	9246	BD157F-BoNT/Di	AADYGDLDNVVVDTPWDY	Z	16	1
BD172	9245	BD166F-H_C_/D	ATVFTGTFITTSDY	X	13	3
BD173	9245	BD166F-H_C_/D	ATVFSGTFTTLNDDY	X	13	3
BD181	9245	BD166F-H_C_/D	AAGGLIWDGSSWTCQSYGMDY	AB	n.a.	3
BD178	9245	H_C_/D	AADLSGSGYGCYDVKANDY	AA	n.a.	3
BD180	9246	BD157F-BoNT/Di	NAEIINSAGRVGY	Y	n.a.	2
BD190	9245	H_C_/D	AKGEL	W	n.a.	C
BD174	9246	BD166F-H_C_/D	NAITSPTG	V	9	X
BD192	9246	H_C_/D	NAITIPMG	V	9	2

^a^ VHH names start with B for BoNT whereas the second letter of the name indicates the BoNT serotype. Unique serial numbers were given during isolation. Underlined VHHs were used for VHH multimerization. ^b^ The recombinant BoNT protein used for phage display selection is indicated. When a VHH is used for its immobilization, the name of the protein is preceded by the name of its capturing VHH. ^c^ The number of different residues between VHHs of the same CDR3 group. ^d^ VHH subfamilies were defined earlier [51]. X indicates VHHs that could not be classified. ^e^ n.a., not applicable.

**Figure 1:**
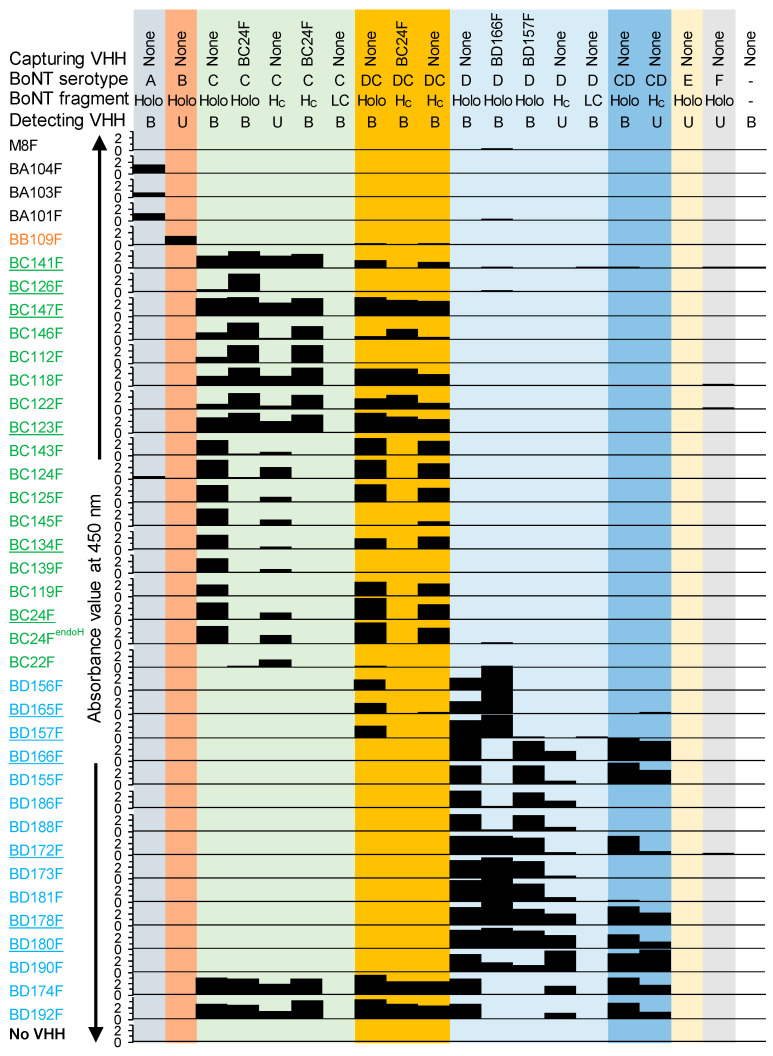
Binding of monomeric VHHs to inactivated recombinant BoNT holotoxins (Holo) or their LC or H_C_ domains in ELISA. In 14 tests, BoNTs or their fragments were directly coated, while in 5 tests, BoNTs or H_C_ domains were captured by a coated VHH. Dashes: control without BoNT protein. The VHHs used to detect coated antigens were biotinylated (B) or unlabeled (U) and detected using streptavidin—HRP conjugate or goat anti-llama IgG—HRP conjugate, respectively. BoNT serotypes are indicated by different background colors. For the ELISAs with BoNT/A holotoxin or H_C_/DC domain the background absorbance values of 0.76 and 0.94, respectively, were subtracted from all values. The other ELISAs had background absorbance values below 0.3 which were not subtracted. BC24F^endoH^ indicates endoglycosidase H-treated BC24F. Underlined VHHs were used for VHH multimerization. M8F is a negative control VHH binding to foot-and-mouth disease virus. Absorbance values range from 0 to 4. The axes do not show the number 4 due to overlap with the number 0 of the graph above.

The binding to inactivated recombinant BoNT proteins of the 36 selected VHHs was analyzed by ELISA (Figure 1). The BoNT proteins encompassed BoNT/Ai, Bi, Ci, CDi, Di, DCi, Ei and Fi holotoxins, H_C_ domains of BoNT/C, CD, D and DC, and LC of BoNT/C and D. Most antigens were directly coated to ELISA plates, but some antigens were captured using VHHs. Preferably biotinylated VHHs were used for BoNT protein detection. However, some ELISAs were performed using unlabeled VHHs when the use of biotinylated VHHs resulted in a high background. The following can be concluded from Figure 1, which is also summarized in Table 2. Endoglycosidase H treatment did not affect BC24F reactivity in ELISA (Figure 1). We therefore used BC24F that was untreated for further experiments. With respect to BoNT serotype specificity, most of the VHHs binding BoNT/C or D did not cross-react to BoNT/A, B, E or F, except BC124F, which shows an absorbance value of 0.484 on BoNT/Ai (Figure 1). This absorbance value is only slightly above the background and thus could represent assay variation rather than BoNT/Ai binding, consistent with the absence of BoNT/Ai binding by BC143F, which belongs to the same CDR3 group. We nevertheless did not select BC124F for further work. Six different types of specificity for the four group III BoNTs were observed. VHHs were specific for either C and DC, C, DC and D, D and CD, D, or recognized all four types (C, DC, D, and CD). With respect to domain specificity, it was observed that none of the VHHs bound the LC domain. Most bound the H_C_ domain (Figure 1), with the exception of VHHs BC126F, BD156F, BD165F and BD157F, which presumably bound the H_N_ domain. Remarkably, BC22F bound the H_C_ domain but not the BoNT/Ci holotoxin. Double antibody sandwich (DAS) ELISAs using BC24F, BD157F or BD166F coating to capture soluble BoNT proteins sometimes did not result in VHH binding while in a direct ELISA using coated BoNT proteins did result in VHH binding. Since each antigenic site is represented only once on BoNTs a DAS ELISA can only give a signal if the two VHHs used bind to different antigenic sites. Therefore, the absence of binding in DAS ELISA while showing binding in direct ELISA suggests binding to the same antigenic site. This was used to map the antigenic sites of the various VHHs (Table 2).

### 2.3. Mapping of Antigenic Sites of Monomeric VHHs

**Figure 2:**
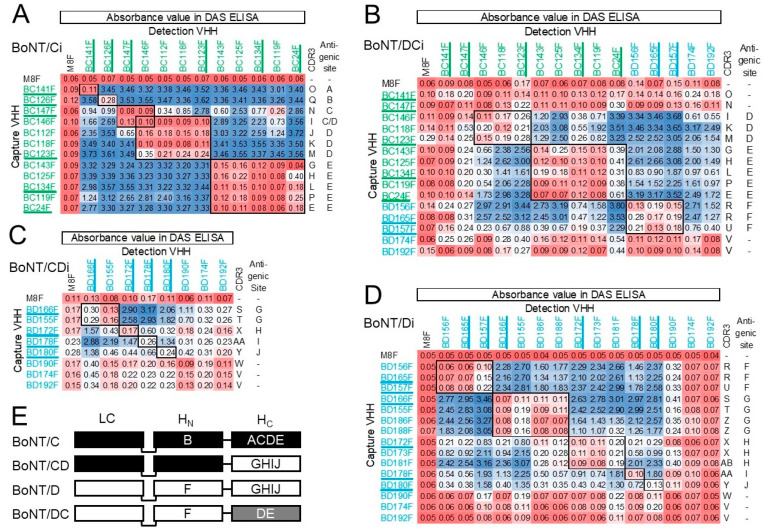
Epitope mapping of VHHs binding to inactivated recombinant BoNTs. DAS ELISAs were completed using unlabeled capture VHH for coating and biotinylated VHH for detection of BoNT/Ci (**A**), BoNT/DCi (**B**), BoNT/CDi (**C**) or BoNT/Di (**D**). Absorbance values obtained in ELISA are indicated using a red/blue coloring scheme. In each panel VHHs are grouped according to the antigenic sites they target, indicated by letters A to J at the right. The CDR3 group of each VHH is also given (A to AB). Underlined VHHs were used for subsequent VHH multimerization. M8F is a negative control (see Figure 1). (**E**) For each BoNT serotype, it is indicated on which domain the identified antigenic sites reside. Domains are colored white, black and grey according to their general sequence homology [16]. Domains of the same color have at least 93% amino acid sequence identity between different BoNT serotypes whereas domains of different color have 40 to 77% sequence identity. The H_C_/DC domain (grey) shows a relatively high 77% identity with H_C_/C, while H_C_/C and H_C_/D domains show only about 40% identity.

CDR3 is crucial for determining antigen-binding specificity [52]. Therefore, VHHs from different CDR3 groups were preferably selected for antigenic site mapping. We used DAS ELISAs with coated unlabeled VHH for capture, and biotinylated VHH for detection. By systematically testing combinations of VHHs for the detection of captured inactivated recombinant BoNTs, antigenic sites of VHHs were mapped (Figure 2). Productive combinations of different VHHs often resulted in absorbance values in the range of 1 to 4. As expected, using the same VHH for capture (unlabeled) and detection (biotinylated) resulted in low ELISA signals (<0.3). Only BC24F on BoNT/DCi gave a relatively high absorbance value of 0.61 using the same VHH twice, which did not prevent epitope mapping since BC24F combinations with other VHHs often gave absorbance values above 2 (Figure 2B).

The VHHs binding BoNT/Ci recognize five different antigenic sites indicated by letters A to E (Figure 2A). BC146F could not be clearly mapped as it gave low absorbance values in combination with VHHs from site C as well as site D, suggesting the recognition site may overlap with both antigenic sites. The VHHs binding BoNT/DCi recognize three independent antigenic sites, D to F (Figure 2B). Sites D and E are defined by VHHs that also bind BoNT/Ci (Figure 2A) and are thus conserved on both toxins. Site F is defined by VHHs that also bind BoNT/Di (Figure 2D). BC141F and BC147F could not be mapped (Figure 2B) as they gave low absorbance values (<0.6) with all other VHHs, indicating inefficient binding to BoNT/DCi. The inefficient binding of these two VHHs in a DAS ELISA setup was not observed using direct coating of BoNT/DCi (Figure 1). BD174F and BD192F, which belong to the same CDR3 group, generally give high absorbance values (1 to 3.2) using biotinylated VHH in combination with unlabeled VHH from sites D, E or F (Figure 2B). This suggests that these VHHs bind to a site that is different from sites D, E and F, which is consistent with their binding to all group III BoNTs (Figure 1). However, when using unlabeled BD174F or BD192F they give low absorbance values (<0.55) with other biotinylated VHHs. Therefore, we did not define them as recognizing a separate antigenic site. The VHHs binding BoNT/Di recognize five antigenic sites, F to J (Figure 2D). BD190F, BD174F and BD192F could not be mapped due to too low absorbance values (<0.4), although they gave high absorbance values in direct ELISA (Figure 1), similar to BC141F and BC147F binding to BoNT/DCi. The VHHs binding BoNT/CDi recognized four antigenic sites, G to J, while BD190F, BD174F and BD192F could again not be mapped (Figure 2C). The recognition of antigenic sites of all other VHHs was consistent with the binding specificity for the four group III BoNT types in direct ELISA (Figure 1, Table 2). Figure 2E summarizes the location of antigenic sites on various BoNT domains.

### 2.4. Construction of VHH Multimers

We next constructed VHH multimers (Figure S2A) to achieve improved BoNT binding affinity by genetic fusion of monomeric VHHs. In total, 12 VHHs (underlined in Table 1 and Table 2) were selected for the construction of multimers based on the following criteria.

- Only VHHs that could be mapped to antigenic sites were included.
- Only VHHs that could be used for the capture of (active) recombinant BoNT/C and D in Endopep-MS were included. Thus, BC112F, BC122F and BC22F were excluded since they were negative in Endopep-MS (Table 2). In the case of BC22F, this is consistent with its inefficient binding of inactivated holotoxin (Figure 1). In the case of the other two VHHs, it is unclear why they were negative in Endopep-MS.
- Five BoNT/C binding VHHs were selected based on their cross-reaction with BoNT/DC in direct ELISAs (Figure 1). However, two of these VHHs, BC141F and BC147F, were later found to bind BoNT/C but not DC in DAS ELISAs (Figure 2) as well as in biolayer interferometry scouting experiments (Figure S3G,H). We thus consider these two VHHs BoNT/C specific. BC126F is also BoNT/C specific.
- Similarly, the six BoNT/D binding VHHs were selected based on their cross-reaction with BoNT/DC (BD157F, BD165F) or CD (four further VHHs).
- Preferentially, VHHs recognizing different antigenic sites were selected. However, BC24F and BC134F both bind BoNT/C antigenic site E while BD165F and BD157F both bind BoNT/D and DC antigenic site F. Here, two VHHs binding the same antigenic site but representing different CDR3 groups were selected to increase chances of producing multimers with improved affinity.

The selected VHHs for BoNT/C and D, six each, were used to construct multimers. In total, 52 VHH multimers comprising 10 trimers and 42 dimers were developed (Figure S2A). They were all composed of VHHs recognizing different antigenic sites. Furthermore, they all bound either BoNT/C and/or DC or bound to BoNT/D and/or CD with at least two of its constituent monomeric VHHs. Based on this predicted multimeric binding to BoNT types we grouped them into multimers binding BoNT/C (22 multimers), DC (15 multimers), D (20 multimers) and CD (16 multimers; Figure S2A). The VHH multimers were produced by yeast expression using flexible 15 amino acid residue gly–ser linkers to join VHH domains and by mutation of the N-terminal 5 residues of C-terminal VHH domains for stability and cloning purposes (Figure S2B). Analysis of yeast-produced VHH multimers by SDS-PAGE (Figure S1C–G) revealed that most VHH multimers migrated at a position corresponding to the expected molecular weight of a VHH dimer (approximately 40 kDa) or trimer (approximately 60 kDa). However, there were two exceptions. Firstly, all multimers containing a BC24 domain had about 50% heterogeneous high molecular weight VHH indicative of N-glycosylation. In the case of three multimers, such N-glycosylation was confirmed by endoglycosidase H treatment (Figure S1D). Secondly, four VHH dimers, BC123BD157F, BD157BC123F, BD157BD166F and BD166BD157F, contained about 10% VHH migrating at about 28 kDa (Figure S1F) indicating degradation of these dimers at a specific site. They all contain a BD157 VHH, suggesting degradation occurs in this VHH, independent if it is present at the N- or C-terminal position of the multimers. Since the band representing these four intact dimers was much stronger than their presumable breakdown products, we included them in further analysis.

### 2.5. Effect of VHH Multimerization on BoNT Affinity

We determined the affinities of all 52 VHH multimers for BoNT binding using biolayer interferometry (Table 3 and Table 4). We measured the affinities of multimers if at least two of the monomeric constituent VHHs could bind the BoNT type (See Figure S2A). However, since we erroneously (see above) assumed that BC141F and BC147F could bind BoNT/DCi, we also measured the affinity for BoNT/DCi of six additional VHH multimers that only contained one constituent VHH binding BoNT/DCi (underlined BoNT/DCi affinity values in Table 3). Furthermore, we measured the affinity for either BoNT/Ci or Di of all monomeric VHHs that were used for VHH multimerization.

The affinities for BoNT/Ci and DCi are shown in Table 3. The monovalent BC147F had a relatively low affinity (high *K_D_*) of 2520 pM as compared to the other five monovalent VHHs binding BoNT/Ci (*K_D_* ≤ 504 pM) due to both a low association rate (*k_a_*) and high dissociation rate (*k_d_*; see also Figure S3A). BC126F had a relatively high affinity of 25 pM due to a low *k_d_*. The multimers had affinities for BoNT/Ci that were comparable or increased compared to their monovalent corresponding VHHs. The fold increase in affinity of the multimers compared to the affinity of the monovalent corresponding VHH with the highest affinity is represented in Table 3. Multimers with C-terminally positioned BC126F, BC134F or BC141F have an increase in affinity of only 0.4 to 2.4-fold suggesting that these C-terminal VHHs are compromised in antigen binding. However, the C-terminal fusions with BC24F, BC123F and especially BC147F have a more substantial increase in affinity of up to >82-fold. This suggests that in these multimers both the N-terminal and the C-terminal VHHs contribute to binding, i.e., these multimers bind multivalently. The increase in affinity of multimers was primarily due to a decreased *k_d_*, that in the case of six multimers reached the detection limit of the biolayer interferometry method (*k_d_* < 0.28 × 10^−5^ 1/s). Seven VHHs (Table 3) were selected for further analysis based on their high affinity for BoNT/Ci.

The affinities of different VHH multimers for BoNT/DCi were 65-719 pM (Table 3), which is generally lower than BoNT/Ci binding affinity. The three VHH multimers with the highest affinities of 65-99 pM were selected for further analysis.

The affinities for BoNT/Di and CDi are shown in Table 4. The monovalent VHHs have a *K_D_* for BoNT/Di of 62 pM for BD166F to 1520 pM for BD180F. The multivalent VHHs have a *K_D_* of <5 to 496 pM. Several multimers did not have increased affinities as compared to the monovalent corresponding VHH with the highest affinity. This was especially true for the BD166 containing multimers, except BD166BD157F. This suggests that in these multimers (except BD166BD157F) the BD166 VHH did not contribute to binding. Five multimers binding BoNT/D with <5 to 79 pM *K_D_* were selected for further analysis. Four of these VHHs also bound BoNT/CDi with high affinity (57–99 pM). These four VHHs comprise one trimer and three dimers and are composed of VHHs BD166, BD172, BD178 or BD180 binding to antigenic sites G, H, I or J (see Table 2).

### 2.6. Effect of 2 M Salt Wash on BoNT Binding

**Figure 3:**
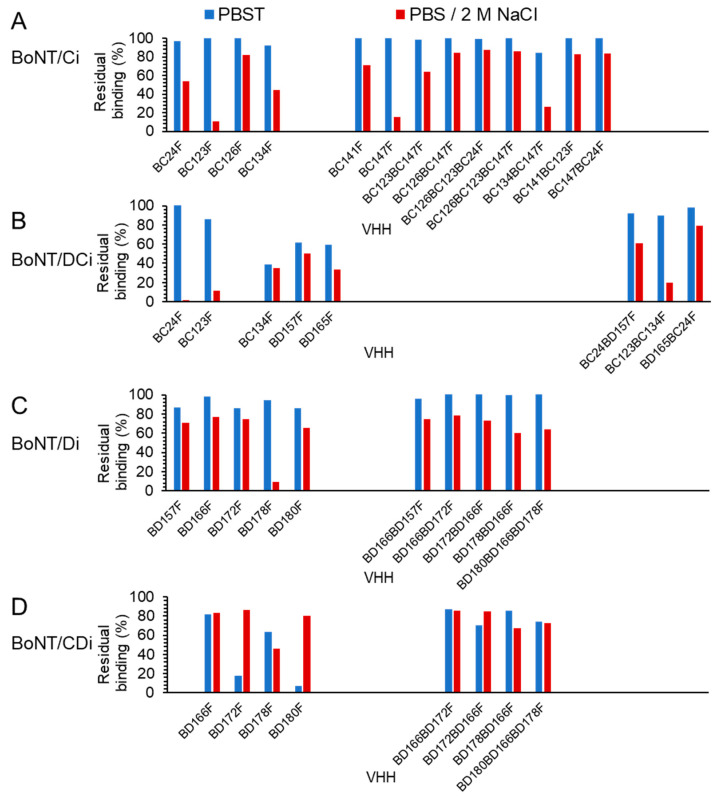
Effect of 2 M NaCl on BoNT dissociation from VHHs. Dissociation of BoNT/Ci (**A**), BoNT/DCi (**B**), BoNT/Di (**C**) or BoNT/CDi (**D**) analyte from sensors loaded with VHHs was measured by biolayer interferometry. All sensorgrams are shown in Figure S4. The percentage residual BoNT signal was measured after the first incubation in PBST and after the second incubation in PBS/2 M NaCl. Gaps were introduced to align identical VHHs in panels (**A**,**B**) as well as (**C**,**D**).

As a high salt wash is often used for samples from complex matrices in routine testing for BoNT, we next investigated the effect of 2 M NaCl on BoNT dissociation from the 15 selected high-affinity multimers (see Table 3 and Table 4). After capturing inactivated recombinant BoNT on sensors loaded with VHHs, the dissociation of BoNT in the absence and presence of 2 M NaCl was measured by biolayer interferometry (see Figure S4 for details). In all assays, the buffer change to PBS/2 M NaCl resulted in a sudden large increase in the signal that rapidly decreased when changing the buffer to HBS-EP (Figure S4), most likely due to buffer effects on biolayer interferometry. BoNTs often dissociated considerably from VHHs during incubation with PBS/2 M NaCl. To assess the effect of high salt, we compared the percentage residual binding during the first incubation in PBS containing 0.05% Tween-20 (PBST) and the second in PBS/2 M NaCl (Figure 3).

Residual BoNT binding to VHH monomers in PBS/2 M NaCl is comparable to that in PBST for some VHHs such as BC126F (Figure 3A) and BD157F, BD166F, BD172F and BD180F (Figure 3C), but strongly decreased as compared to PBST for other monomers such as BC24F (Figure 3B), BC123F (Figure 3A,B) and BD178F (Figure 3C). Residual BoNT binding in PBS/2 M NaCl to VHH multimers is 64–78% (BoNT/Ci), 20–79% (BoNT/DCi) or 60–86% (BoNT/Di and CDi) and equal or higher as compared to their corresponding VHH monomers for all four BoNT types (Figure 3), consistent with their higher affinity for BoNT in PBST (Table 3 and Table 4).

### 2.7. Detection of Native BoNTs with Use of VHH Multimers in Endopep-MS

**Figure 4:**
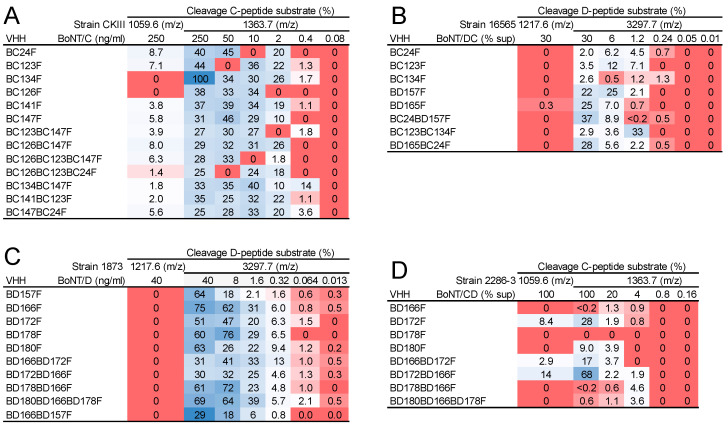
Endopep-MS analysis using VHHs. Native BoNT/C (**A**), BoNT/DC (**B**), BoNT/D (**C**) or BoNT/CD (**D**) obtained from *C. botulinum* culture supernatant was subjected to Endopep-MS in 5-fold dilution series using various monomeric and multimeric VHHs. The multimeric VHHs were selected based on high affinity. For BoNT/DC and CD, the percentage supernatant (% sup) is indicated since the toxin concentration is unknown. MS analysis was completed after Endopep reactions for 24 h (**C**) or 48 h (**A**,**B**,**D**). Cleavage of substrate peptides is presented as the percentage ratio between the peak surface area of the specific cleavage product shown and the sum of the peak surface areas of the NT product, CT product and substrate peptide. Signal differences are visualized by red-blue coloring.

We investigated whether the 15 selected high-affinity multimers (see Table 3 and Table 4) were suitable for immunocapture of native BoNTs and their detection in Endopep-MS, in comparison with the corresponding monovalent VHHs (Figure 4). Native BoNT/C, DC, D, and CD were produced by culturing *C. botulinum* strains. The amount of BoNT/C and D could be quantified with DAS-ELISAs using recombinant inactivated BoNT proteins as standard. The BoNT/DC and CD proteins could not be quantified by DAS-ELISA due to a too low ELISA signal. The amounts of BoNT/C and D used in Endopep-MS were thus expressed as ng/mL, whereas the amount of BoNT/CD and DC was expressed as percentage culture supernatant tested (Figure 4). For each BoNT type, toxins were titrated in fivefold dilutions in order to compare the selected VHHs for their limit of detection in Endopep-MS. BoNT was detected based on the identification of the large cleavage products, which are 1363.7 *m*/*z* for the BoNT/C peptide substrate and 3297.7 *m*/*z* for the BoNT/D peptide substrate. Based on the relative peak surface area, the small cleavage products of, respectively, 1059.6 *m*/*z* and 1217.6 *m*/*z* for BoNT/C and BoNT/D peptide substrates were often of much lower intensity than the large cleavage products. This was especially evident for cleavage of the BoNT/D substrate as shown for the highest BoNT concentration analyzed (Figure 4B,C), but also for cleavage of the BoNT/C peptide (Figure 4A,D). We therefore further analyzed Endopep-MS data based on the large cleavage products only.

All VHH monomers and multimers were able to capture the BoNT types that they were expected to bind based on ELISA (Figure 1), with the exception of BD178F, which detected BoNT/D but failed to detect BoNT/CD even at the highest amount of toxin used, as demonstrated by Endopep-MS (Figure 4). No major differences in sensitivity were observed between monomeric, dimeric or trimeric VHHs (Figure 4A). The sensitivity of BoNT/DC detection was similarly not increased by VHH multimerization (Figure 4B). BoNT/D could be detected down to the lowest concentration of 0.013 ng/mL, using both monomers and multimers. Here, the BD166BD157F and BD178BD166F multimers were less sensitive than the other multimers, and less sensitive than, e.g., the constituent VHH BD166F (Figure 4C). The sensitivity of BoNT/CD detection was also not unambiguously increased by VHH multimerization (Figure 4D).

## 3. Discussion

We isolated 34 novel VHHs against BoNTs with a focus on group III BoNTs. The VHHs were selected against BoNT/Ci and Di by phage display. Some VHHs were shown to cross-react with BoNT/DCi or CDi mosaic variants. During selections we used, next to the holotoxins, the H_C_ domains since for endopeptidase-based tests it is important to have a fully active LC. VHHs binding H_C_ are least likely to interfere with Endopep-MS because it is located distant from the LC domain (Figure 2E) that carries the enzymatic activity. The use of antibodies against H_C_ was recommended after the screening of mAbs for use in Endopep-MS showed inhibition of BoNT activity by LC binding mAbs [53]. Furthermore, BoNTs in complex matrices that are proteolytically cleaved in the H_N_ domain cannot be detected by Endopep-MS after immunocapture specific for H_C_ as the LC-carrying activity is not captured. Thus, the use of H_C_-specific capture antibodies reduces false positives in Endopep-MS as compared to LC or H_N_-specific capture antibodies [24,54]. However, BC22F binds a site only available in the H_C_ protein but not in BoNT/Ci holotoxin. Presumably, this VHH binds to a site close to the H_C_/C N-terminus that is not accessible in the BoNT holotoxin due to steric hindrance by the H_N_ domain, as suggested earlier [30].

The affinity of VHH multimers generated in this study for BoNT/DCi is generally lower than for BoNT/Ci. This is presumably due to the specificity of most monomers for H_C_/C, which shows only about 77% identity with H_C_/DC [7]. Consistent with this conclusion VHH multimers showed much more similar affinities for BoNT/Di and CDi, of which the sequence identity of the H_C_ domains is much higher.

BD174F and BD192F, both found during phage display selection with H_C_/D, were the only two VHHs that bind both BoNT/Ci and Di, and thus also the mosaic variants. None of the other VHHs recognized both BoNT/Ci and Di. MAbs that recognize both BoNT/C and D were isolated earlier and mostly bound H_N_ [29,55], which has a region with high sequence homology of BoNT/C and D [7]. Furthermore, an mAb that recognized BoNT/A, B, E and F was also shown to bind to H_N_ [11]. However, VHHs BD174F and BD192F were found to bind H_C_ and thus recognize a common epitope in group III H_C_ domains. Since these VHHs did not perform well in DAS-ELISA their antigenic site(s) could not be mapped by epitope binning. A further two VHHs, BC141F and BC147F, similarly did not bind well to BoNT/DCi in DAS ELISA as well as in biolayer interferometry assays, although they did bind to directly coated BoNT/DCi. This could indicate an epitope that is altered or blocked upon direct coating of BoNT. Such effects of direct coating of antigens by passive adsorption to polystyrene on antigenicity have been observed earlier [56,57]. In the case of BC141F and BC147F, the relatively low affinity for BoNT/Ci (*K_D_* > 0.5 nM), which probably is even lower for BoNT/DCi, could also cause reduced binding to soluble BoNT/DCi.

The VHHs that performed well in DAS-ELISA recognized independent antigenic sites A–E for BoNT/Ci and sites F-J for BoNT/Di. At least 20 independent antigenic sites were identified on TeNT [58], which is structurally highly similar to BoNT, and the 61 kDa ricin toxin [59]. The lower number of antigenic sites identified by our panel of VHHs could be due to the high proportion of VHHs binding H_C_, which encompasses four out of five antigenic sites recognized for both BoNT/C and D.

Oligoclonal mixtures of recombinant antibodies recognizing different antigenic sites were earlier demonstrated to increase the sensitivity of BoNT/A detection [60]. However, the multimerization of VHHs was proven more effective than making equimolar mixtures of VHHs for increasing affinity, as shown for Shiga toxin [44] and BoNT [46]. Therefore, using a set of 12 monomeric VHHs, we chose to produce a broad panel of 52 VHH multimers composed of 2 or 3 constituent VHH domains that each recognize an independent antigenic site. Using biolayer interferometry we identified a number of VHH multimers with increased affinity (avidity) as compared to the monovalent corresponding VHH with highest affinity (Table 3 and Table 4). However, some VHH multimers were found to have lower affinities than the optimal corresponding monomers. This was especially apparent for many multimers against BoNT/D containing BD166, which has the highest affinity (*K_D_* = 62 pM) among the BoNT/D binding VHHs. Since the N-terminus of a VHH is close to the antigen-binding site, the genetic fusion of a VHH can affect antigen binding, especially the C-terminal VHH due to steric hindrance by the N-terminal VHH [37]. Furthermore, mutations Q1E and Q5V of the C-terminal VHH (see Figure S2) could also affect antigen binding. These two mechanisms can explain compromised antigen binding of the C-terminal VHH domain but not the N-terminal VHH domain in a multimer. However, many multimers containing an N-terminal BD166 domain also show lower affinities than the BD166F monomer (Table 4). This could be due to prior binding by C-terminal VHH(s) to BoNT affecting binding by the BD166 domain because the two antigenic sites are not properly positioned to allow simultaneous binding of the linked VHH domains.

It is generally assumed that the increased affinity of heterodimeric antibodies is due to the binding of one antigen-binding domain resulting in large increases in “target residence time”, which can lead to rapid rebinding by other domains upon dissociation of a single antigen-binding domain [61]. However, such a model only applies if both antigen-binding domains recognize epitopes that are suitably oriented to allow simultaneous binding of two genetically linked antibody domains. Long flexible linkers such as the 15 amino acid (GGGGS)_3_ linker used here are generally employed for VHH multimerization to increase the conformational flexibility required for such simultaneous binding to different epitopes on a single antigen molecule. We assumed that such simultaneous binding is more often seen for multimers that recognize different toxin domains because conformational flexibility between domains more likely results in properly oriented epitopes. Therefore, we also produced multimers composed of BD157 or BD165 domains, that bind antigenic site F on H_N_, in combination with VHHs binding H_C_ domains for both BoNT/DCi (Table 3) and BoNT/Di or CDi (Table 4). However, we did not clearly observe higher binding affinities of VHH multimers containing VHHs that bind to different toxin domains as compared to multimers that bind different antigenic sites on the same (H_C_) domain. For multimers with BC126, which is assumed to bind H_N_/C, such improved binding is more difficult to demonstrate since BC126F already has a high affinity of 25 pM. Furthermore, the H_N_ binding of BC126F is only based on the absence of binding to recombinant H_C_ in direct ELISA and is not confirmed by specificity for BoNT types in ELISAs, as was the case for BD157F and BD165F. The exact epitopes bound by our VHHs were not further determined, beyond the specificity for BoNT domains. More detailed knowledge of epitopes bound by X-ray crystallography was used earlier for the structure-based rational design of VHH multimers with potent BoNT neutralization for therapeutic purposes [33].

Fifteen multimers were found to have a high affinity (*K_D_* < 0.1 nM; Table 3 and Table 4). They were found suitable for binding to BoNTs in the presence of 2 M NaCl, which is often used as a wash step of samples immunocaptured from complex matrices to remove aspecific proteases prior to Endopep-MS [13,16,25]. Using high salt, VHH multimers generally showed decreased dissociation as compared to VHH monomers, consistent with their increased affinity. The 15 high-affinity VHH multimers were found suitable for the detection of the 4 group III BoNTs produced from bacterial culture in Endopep-MS. Unexpectedly, the limit of detection (LOD) in these assays of VHH multimers was not higher than their corresponding monomers. This could be caused by not all constituent VHHs in a multimer contributing to BoNT binding, due to variation in BoNTs from different strains [62], which often occurs with mosaic variants [63]. However, other (unidentified) reasons for multimers with increased affinity not resulting in an increased sensitivity of Endopep-MS, cannot be excluded.

Bjornstad et al. [14] developed an excellent Endopep-MS test design to discriminate the four group III BoNT-types based on different combinations of specificity of the capture antibody and specificity of BoNT proteolytic activity. A similar approach can be achieved if two or more VHH multimers are used that specifically bind either BoNT/C and DC or BoNT/D and CD, in combination with different peptide substrates for LC/C and LC/D. However, the three multimers BC24BD157F, BD165FBC24F and BD166BD157F are not suitable for such discrimination of all four group III BoNTs in Endopep-MS since they contain the VHHs BD165 or BD157 that both recognize site F on H_N_ of BoNT/D and DC (Figure 2E). The above considerations will be taken into account in our future studies to determine which high-affinity multimers are most suited for diagnosing BoNT in clinical samples using Endopep-MS.

## 4. Conclusions

Using novel VHHs against group III BoNTs, 52 VHH multimers were produced for high-affinity (avidity) BoNT binding due to the multimeric binding of constituent VHHs to adjacent antigenic sites on a single BoNT molecule. Using an empirical approach several multimers were obtained that, as compared to their corresponding VHH monomers, showed increased affinity as well as better binding using high salt washes that are commonly used for diagnosing BoNT in complex matrices. These high-affinity multimers proved suitable for the detection of native group III BoNTs in Endopep-MS. Further validation of these high-affinity multimers in Endopep-MS in complex matrices is needed to confirm that multimers outperform monomers. In addition to application in diagnostic tests, the monomeric and multimeric VHHs could also provide a basis for the generation of VHH-based therapeutics for animal treatment against intoxication with group III BoNTs. Such therapeutics are also relevant for humans, where group III BoNTs pose a biothreat by those of ill intent.

## 5. Materials and Methods

### 5.1. Recombinant BoNT Proteins

Recombinant BoNT proteins (Table 5) were obtained from Toxogen GmbH (Hanover, Germany). They were produced in *E. coli* according to previously described methods, including limited thrombin proteolysis to activate BoNT holotoxins [16,64,65]. Inactivated recombinant BoNTs contained 3 mutations in the light chain for toxin inactivation and are referred to by the suffix i.

### 5.2. Toxin Production by Bacterial Cell Culture

Bacterial cell culture supernatants were prepared from several *C. botulinum* strains that were obtained from Dutch clinical and food samples (Table 6) that were described earlier [66]. The BoNT type of these strains was confirmed by neurotoxin gene profiling using the GeneDisc cycler platform [67]. Furthermore, the identity of the BoNT LC was confirmed based on its substrate specificity in Endopep-MS. The strains were inoculated from glycerol stocks in 5 mL Cardella medium (2% N-Z-Case, 4% proteose peptone, 2% yeast extract, 1% glucose, pH 7.8) and grown to stationary phase during 3 days at 30 °C under anaerobic conditions. Supernatants were collected by centrifugation for 5 min at 12,000× *g* and 4 °C and stored aliquoted at −20 °C. BoNT/C and D concentrations in supernatant were determined by DAS ELISA (Table 6) using VHHs as described below (Section 5.6). For native BoNT/D, production strain 1873 was used. The BoNT gene sequence of this strain is identical to that of strain BVD/-3 [16], which is used for production of recombinant BoNT/Di. Therefore, it is likely that the procedure used for quantifying BoNT/D from strain 1873 with recombinant BoNT/Di as standard gives a correct result. For production of native BoNT/C, strain CKIII was used while recombinant BoNT/Ci that was used as standard, was produced by strain 468C phage C-St. Since the BoNT gene sequence of strain CKIII is unknown, it cannot be excluded that differences between the sequences of BoNT/C from CKIII and the recombinant BoNT/Ci standard could have affected antigenicity and thus quantification.

### 5.3. Phage Display Selection of BoNT Binding VHHs

We recently described the immunization of llamas with inactivated foot-and-mouth disease virus [68]. These 2 llamas (9245 and 9246) were also immunized with recombinant inactivated BoNT proteins (Table 5), using 25 µg of each toxin per immunization per llama. Llama 9245 was immunized 3 times with 3-week intervals with a mixture of BoNT/Ai, Bi, Ci, Di, Ei and Fi. Llama 9246 was first immunized with BoNT/Ci, 3 weeks later with BoNT/Di and a further 3 weeks later again with BoNT/Ci.

Phage display selections of phage libraries in plasmid pRL144 were performed by two consecutive rounds of biopanning with simultaneous phage ELISA to monitor the panning progress, as described [68]. Briefly, biopanning was performed on recombinant H_C_ domain or inactivated BoNT holotoxins captured on streptactin plates (Thermo Fisher Scientific, Rockford, IL, USA) or directly coated to high-binding polystyrene 96-well plates (Greiner, Solingen, Germany). Furthermore, we used the following VHHs for capture of recombinant antigens during biopanning: BC24F for H_C_/C or BoNT/Ci, BD166F for H_C_/D and BD157F for BoNT/Di. Recombinant BoNTs were used at concentrations of 0.1 or 1 µg/mL during panning. After the second panning round, phages were transduced to *Escherichia coli* TG1 cells, and plated for single colonies. Several hundreds of clones were then induced for production of VHH using isopropyl β-d-thiogalactopyranoside. Periplasmic extracts containing soluble VHH were then tested for binding to BoNTs as described below (Section 5.6).

### 5.4. Sequence Analysis

Sequencing of VHHs was completed as described previously [68]. The deduced VHH amino acid sequences were aligned according to the IMGT system [69] for alignment, numbering, and CDR definition of immunoglobulins. VHHs were classified into subfamilies as defined earlier [51] and into CDR3 groups based on at least 50% CDR3 sequence identity. Potential N-glycosylation sites were defined as Asn-X-Ser/Thr, where X can be any amino acid residue, except Pro.

### 5.5. Yeast Production of Monomeric and Multimeric VHHs

The novel VHHs isolated in this study were inserted as PstI-BstEII fragments into plasmid pRL188. This results in the production of mature VHHs starting with sequence QVQLQ and C-terminal fusion to the llama heavy-chain antibody long hinge region containing a single cysteine enabling partial dimerization, which is suitable for direct VHH coating [47]. Using the resulting pRL188-derived plasmids, VHH monomers were expressed in *Saccharomyces cerevisiae* strain SU51, purified by IMAC and biotinylated at a weight ratio of protein to biotin of 5 using amine-reactive sulfo-N-hydroxysuccinimide-LC-biotin as previously described [47]. Such yeast-produced VHHs are indicated by the suffix F. The VHHs C22 and C24, which were isolated earlier [30], were similarly expressed in yeast using plasmid pRL188. However, SacI and BstEII restriction sites were used for their cloning into pRL188 to mutate the mature VHH N-terminal amino acid sequence QVKLQA [30] to EVQLVE, to increase stability and production levels [70]. We refer to these yeast-produced VHHs as BC22F and BC24F.

In total, 52 VHH multimers were generated and produced using plasmid pRL188, as described above for monomers. The multimeric VHHs generated are shown in Figure S2A while the cloning scheme is shown in Figure S2B. Synthetic gene fragments (Genscript Corporation, Piscataway, NJ, USA) encoding an N-terminal VHH cloning site with PstI and BstEII sites, the C-terminus of the N-terminal VHH ending at VTVSS, a (GGGGS)_3_ linker, a C-terminal VHH starting with EVQLV and the long-hinge region with his6 tag as present in pRL188 [47] were first inserted into the PstI and HindIII sites of pRL188. These fragments start with sequence 5′-CTGCAGGAGTCATAATGAGGGACCCAGGTCACCGTCTCCTCAGGTGGAGGCGGTTCAGGCGGAGGTGGCTCTGGCGGTGGCGGAAGTGAAGTTCAATTGGTT (PstI and BstEII sites underlined; (GGGGS)3 encoding region double underlined). In total, 12 pRL188-derived (acceptor) plasmids with different C-terminal VHHs were generated. These acceptor plasmids had unique PstI and BstEII sites for insertion of N-terminal VHHs due to mutation of the PstI and BstEII sites of the C-terminal VHH. Subsequently, 12 synthetic gene fragments encoding different N-terminal VHHs were inserted into the PstI and BstEII sites of specific acceptor plasmids to generate VHH dimers. Furthermore, 6 synthetic PstI-BstEII gene fragments encoding fusions of different VHHs linked by a (GGGGS)_3_ linker were inserted into specific acceptor plasmids to generate 10 VHH trimers. These synthetic fragments had silent mutation of the BstEII site of the N-terminal VHH while the C-terminal VHH (middle VHH in trimer) started with sequence EVQLV, removing the PstI site. VHH multimers are named according to their corresponding VHH monomers.

VHHs were analyzed by reducing SDS-PAGE using precast gels (Novex, San Diego, CA, USA) and stained using Gelcode Blue reagent (Thermo Fisher Scientific). VHHs were deglycosylated using endoglycosidase H (New England Biolabs, Ipswich, MA, USA) according to the manufacturer’s instructions.

### 5.6. ELISAs

Four different ELISA procedures were used that were all described in detail recently [68]. Here, procedures adapted for BoNT proteins are described in short. The basic procedure that was used in all ELISAs involved coating high-binding polystyrene 96-well plates (Greiner) with 0.1–2 μg/mL of unlabeled VHH or BoNT protein in 50 mM carbonate/bicarbonate buffer, pH 9.6 (coating buffer). The VHH-coated plates were subsequently incubated with BoNT proteins in either ELISA buffer (1% skimmed milk; 0.05% Tween-20; 0.5 M NaCl; 2.7 mM KCl; 2.8 mM KH_2_PO_4_; 8.1 mM Na_2_HPO_4_; pH 7.4) or PBS containing 1% skimmed milk and 0.05% Tween-20 (PBSTM). Plates containing either directly coated BoNT or VHH-captured BoNT proteins were next incubated with either phage-displayed VHH in PBSTM, *E. coli*-produced VHH in PBSTM, or yeast-produced VHH in ELISA buffer, and subsequently with a suitable specific horse radish peroxidase (HRP) conjugate, using the same buffer as used for incubation with BoNTs (ELISA buffer or PBSTM). The HRP activity was subsequently detected by staining with 1-Step™ Ultra TMB-ELISA Substrate (Thermo Fisher Scientific), stopped by addition of sulfuric acid, and quantified by measuring the absorbance at 450 nm (A450) using a SpectraMax ABS plus ELISA reader (Thermo Fisher Scientific).

For phage ELISAs, BoNT proteins (1 μg/mL) were immobilized to plates either by direct coating or using prior coating of low concentrations of VHH (0.1–1 μg/mL) followed by capture of BoNT proteins in PBSTM. Plates were then incubated with phages and bound phages were detected by incubation with HRP-conjugated mAb against the M13 p8 coat protein (GE Healthcare, Little Chalfont, UK) in PBSTM.

For ELISA analysis of *E. coli*-produced soluble VHH, BoNT proteins (1–2 μg/mL) were either directly coated or captured on ELISA plates coated with unlabeled VHHs (1 μg/mL). Bound BoNT proteins were detected by incubation with 10-fold diluted *E. coli*-produced soluble VHH, which contains a myc-tag, and HRP-conjugated mAb 9E10 against the c-myc tag (Roche Applied Science, Penzberg, Germany). Similar ELISAs for detection of coated BoNT proteins were also completed using yeast-produced VHHs that were either biotinylated or unlabeled. Bound biotinylated VHH was detected with 1 μg/mL HRP-conjugated streptavidin (Jackson ImmunoResearch Laboratories Inc., West Grove, PA, USA) while bound unlabeled VHH was detected using a 10,000-fold diluted polyclonal goat anti-llama IgG-HRP conjugate (Bethyl Laboratories, Montgomery, TX, USA).

BoNT proteins in culture supernatants were quantified using 2 DAS ELISAs. The BoNT/C ELISA was completed using unlabeled BC24F coating, recombinant BoNT/C standard and biotinylated BC123F for detection of captured BoNT. The BoNT/D ELISA employed BD157F coating, recombinant BoNT/D standard and biotinylated BD166F. Both ELISAs were subsequently completed as follows. Plates were coated with 0.5 μg/mL of unlabeled yeast-produced VHH and then incubated with ELISA buffer containing 4-fold dilution series of both *C. botulinum* culture supernatants (Table 6) as well as standards of recombinant active BoNT proteins (Table 5) at 1 μg/mL starting concentration. Plates were next incubated with 0.25 μg/mL of biotinylated VHH. Bound biotinylated VHH was detected with HRP-conjugated streptavidin (Jackson ImmunoResearch Laboratories Inc., West Grove, PA, USA). A four-parameter logistic curve was fitted to absorbance and BoNT protein concentrations of standards. The BoNT protein concentration in unknown samples was then determined by interpolation.

Antigenic sites were mapped by DAS-ELISAs using different combinations of unlabeled (coating) and biotinylated VHHs. Since each antigenic site on the BoNT protein is only represented once, BoNTs can only be detected in DAS-ELISAs if the unlabeled and biotinylated VHHs recognize different antigenic sites. We made 4 separate epitope maps using recombinant BoNT/Ci, Di, CDi or DCi (Table 5). For each epitope map we included at least 1 VHH representative of each CDR3 group since VHHs from the same CDR3 group in general bind the same antigenic site [68]. The DAS-ELISAs were completed as described above, using ELISA buffer. Briefly, plates coated with 0.5 µg/mL unlabeled VHHs were incubated with 1 µg/mL inactivated recombinant BoNT. Bound BoNT was detected using incubation with 0.25 µg/mL biotinylated VHHs. Control incubations with biotinylated versions of the unlabeled (coating) VHHs were always included. Bound biotinylated VHH was detected as described above. The A450 values obtained after TMB staining were ordered in a matrix of unlabeled versus biotinylated VHHs where VHHs recognizing the same antigenic site were grouped together.

### 5.7. Biolayer Interferometry Measurements

The Octet Red96 System (Sartorius, Bohemia, NY, USA) was used for affinity measurements based on biolayer interferometry. An assay temperature of 30 °C was used, with PBST as kinetics buffer in all steps of each assay. High precision streptavidin (SAX)-sensors (Sartorius) were hydrated and subsequently loaded with biotinylated VHHs (0.05 to 3 µg/mL) for 300 s, followed by 300 s incubation with kinetics buffer to establish the baseline. Next, association of BoNT was studied for 300 s using serial dilutions of recombinant BoNT proteins (most VHHs) or 900 s (BC47F only). Finally, dissociation in kinetics buffer was studied for 600 or 1800 s. For high-affinity multimers, we used a dissociation time of 1800 s. The concentrations of VHHs and BoNT proteins were optimized for affinity measurements prior to the experiments. A reference sensor without BoNT proteins was included to correct for baseline drift. The association (*k_a_*) and dissociation (*k_d_*) rate constants were determined by global fitting of the association and dissociation phases of a series of BoNT protein concentrations. The mathematical model used assumes a 1:1 stoichiometry, fitting only one BoNT protein binding to one VHH. Unless otherwise mentioned, the observed fits always had a full R^2^ > 0.98. The equilibrium dissociation constant (*K_D_*), a measure of affinity, was calculated as the ratio of *k_d_* and *k_a_*. The Octet Analysis Studio v12.2 software (Sartorius) was used for data analysis. For very slowly dissociating interactions the *k_d_* was set at 0.28 × 10^−5^ 1/s, assuming a 0.5% decrease in signal during an 1800 s dissociation phase would be detectable and according to the formula *k_d_* < −ln(0.995)/1800 [71].

The effect of a high salt wash on BoNT binding was also determined by biolayer interferometry. Briefly, biotinylated VHHs were loaded on SAX biosensors and 20 nM inactivated recombinant BoNT analyte was captured. BoNT dissociation was subsequently measured during 4 subsequent incubations of 600 s in PBST, PBS/2 M NaCl, HBS-EP (10 mM HEPES pH 7.4, 0.15 M NaCl, 3 mM EDTA, 0.005% Surfactant P20) and PBST. The percentage residual BoNT signal from 30 s after start of dissociation and 30 s before end of dissociation was then determined for both the first incubation in PBST and the subsequent incubation in PBS/2 M NaCl, and the percentage of the signal remaining after each incubation (residual binding) was determined.

### 5.8. Endopep-MS Analysis

#### 5.8.1. Immunocapture of BoNTs from Samples

Recombinant (Table 5) or native (Table 6) BoNTs were extracted from samples using biotinylated VHHs coupled to Dynabeads M-280 Streptavidin beads (Thermo Fisher Scientific) that were separated from liquid using a DynaMag magnet (Thermo Fisher Scientific). To this end, the magnetic beads (250 μL) were first washed twice with 1 mL HBS-EP in Protein LoBind Eppendorf tubes, before suspension in 1 mL HBS-EP containing 5 μg/mL of biotinylated VHH or mAb. After 1 h incubation at RT while rotating at 30 rpm, the beads were washed twice with 1 mL HBS-EP and suspended in 250 μL HBS-EP.

A total of 20 μL of vortexed antibody-coupled bead suspension was used to extract BoNTs from 250 µL samples in HBS-EP, also containing 10% FCS and 2 µL protease inhibitor cocktail (Sigma Aldrich, St. Louis, MO, USA) to inhibit the aspecific proteolytic activity in the sample. The mixture of beads and sample was rotated at 30 rpm for 1–2 h at RT to allow toxin binding. Thereafter, the magnetic beads were washed twice with 1 mL HBS-EP and once with 150 μL water. The beads were finally released in 20 μL Endopep reaction buffer.

#### 5.8.2. Endopep-MS

Endopep-MS analysis to determine BoNT/C or BoNT/D proteolytical activity using specific peptide substrates was performed as described earlier [13]. The 20 µL Endopep reaction buffer (10 mM DTT, 200 μM ZnCl_2_, 1.0 mg/mL BSA, and 50 μM of BoNT/C or BoNT/D peptide substrate in 20 mM HEPES buffer pH 7.3) containing the beads with toxin extracts was transferred to a new Protein LoBind Eppendorf tube, vortexed and incubated for 24 h or 48 h at 37 °C. Peptides (HPLC-purified, purity > 95%) were synthesized by Pepscan (Lelystad, The Netherlands). The peptide Ac-VKYNIDEAQNKAS-Ornithin-MGIRRR-NH_2_ (*m*/*z* 2405.3) based on the human SNAP-25 sequence and further optimized for BoNT/C detection in Endopep-MS [26] was used for BoNT/C and BoNT/CD detection. It yields the NT Ac-VKYNIDEAQNK (*m*/*z* 1363.7) and CT AS-Ornitin-MGIRRR-NH_2_ (*m*/*z* 1059.6) cleavage products. The peptide H_2_N-LQQTQAQVDEVVDIMRVNVDKVLERDQKLSELDDRADAL-OH (*m*/*z* 4498) based on the sequence of the VAMP-2 protein was used for BoNT/D and /DC detection. It yields the NT H_2_N-LQQTQAQVDEVVDIMRVNVDKVLERDQK (*m*/*z* 3297.7) and CT LSELDDRADAL-OH (*m*/*z* 1217.6) cleavage products.

#### 5.8.3. Mass Spectrometry Analysis

After incubation for 24 h or 48 h, the substrate peptide cleavage products were detected by MALDI-TOF MS using an MALDI Biotyper 3.1 (Bruker Daltonics, Bremen, Germany). A sample was spotted onto an MSP-96 target polished stainless steel BC MALDI target plate 3 times using 1.0 μL for each spot. Afterward, the spots were fixed by 1 µL 10 μg/μL α-cyano-4-hydroxycinnamic acid (Bruker Daltonics) in 50% acetonitrile/ 47.5% water/2.5% trifluoroacetic acid (Sigma-Aldrich, St. Louis, MI, USA). The MALDI parameters from the MALDI Biotyper 3.1 were the same as described earlier [14]. In short, the instrument was operated in MS resolution mode in a mass range of 100–8000 *m*/*z*. The laser firing power was 25% of its maximum. Each spectrum was the result of 80 satisfactory laser shots per raster spot. The instrument was calibrated using the Peptide Calibration Standard II (Bruker Daltonics, Billerica, MA, USA) according to the protocol provided by the supplier. Spectra were processed with background subtraction and the automatic peak detection option in the Compass Flex control RTC 3.1 software. The signal-to-noise (S/N) threshold is defined by the mass peak above its baseline relative to the standard deviation of the noise. Only peaks with an S/N value above 3 are identified by the software. The BoNT activity was evaluated by measurement of the cleavage products for BoNT/C (*m*/*z* 1059.6 and *m*/*z* 1363.7) and BoNT/D (*m*/*z* 3297.7 and *m*/*z* 1217.6). The relative peak surface areas as calculated by the software for the substrate and two product peptides were then summarized and set at 100%. Endopep-MS results were either presented as positive/negative based on detection of one of the cleavage products (S/N > 3), or by indicating the relative peak surface area of the cleavage products.

## Figures and Tables

**Table 2:** Binding to recombinant BoNTs of 34 novel VHHs and 2 VHHs published earlier.

		ELISA Binding ^b^		Antigenic Sites ^c^				Endopep-MS, 2 ng Recombinant BoNT	
VHH ^a^	CDR3 Group	H_C_	Inactivated BoNTs	C	DC	D	CD	BoNT Type	Positive (S/N > 3)
BoNT/A									
BA104F	A	ND ^d^	A	ND	ND	ND	ND	BoNT/A	Y
BA103F	B	ND	A	ND	ND	ND	ND	BoNT/A	Y
BA101F	C	ND	A	ND	ND	ND	ND	BoNT/A	Y
BoNT/B									
BB109F	D	ND	B	ND	ND	ND	ND	BoNT/B	Y
BoNT/C									
BC141F	O	Y	C, DC ^e^	A	-	ND	ND	BoNT/C	Y
BC126F	Q	N	C	B	ND	ND	ND	BoNT/C	Y
BC147F	N	Y	C, DC ^e^	C	-	ND	ND	BoNT/C	Y
BC146F	I	Y	C, DC	C, D	D	ND	ND	BoNT/C	Y
BC112F	J	Y	C	D	ND	ND	ND	BoNT/C	N
BC118F	K	Y	C, DC	D	D	ND	ND	BoNT/C	Y
BC122F	K	Y	C, DC	ND	ND	ND	ND	BoNT/C	N
BC123F	M	Y	C, DC	D	D	ND	ND	BoNT/C	Y
BC143F	G	Y	C, DC	E	E	ND	ND	BoNT/C	Y
BC124F	G	Y	C, DC	ND	ND	ND	ND	BoNT/C	Y
BC125F	H	Y	C, DC	E	E	ND	ND	BoNT/C	Y
BC145F	H	Y	C, DC	ND	ND	ND	ND	BoNT/C	Y
BC134F	L	Y	C, DC	E	E	ND	ND	BoNT/C	Y
BC139F	L	Y	C	ND	ND	ND	ND	BoNT/C	Y
BC119F	P	Y	C, DC	E	E	ND	ND	BoNT/C	Y
BC24F	E	Y	C, DC	E	E	ND	ND	BoNT/C	Y
BC22F	F	Y ^f^	C	ND	ND	ND	ND	BoNT/C	N
BoNT/D									
BD156F	R	N	DC, D	ND	F	F	ND	BoNT/D	Y
BD165F	R	N	DC, D	ND	F	F	ND	BoNT/D	Y
BD157F	U	N	DC, D	ND	F	F	ND	BoNT/D	Y
BD166F	S	Y	D, CD	ND	ND	G	G	BoNT/D	Y
BD155F	T	Y	D, CD	ND	ND	G	G	BoNT/D	Y
BD186F	Z	Y	D	ND	ND	G	ND	BoNT/D	Y
BD188F	Z	Y	D	ND	ND	G	ND	BoNT/D	Y
BD172F	X	Y	D, CD	ND	ND	H	H	BoNT/D	Y
BD173F	X	Y	D	ND	ND	H	ND	BoNT/D	Y
BD181F	AB	Y	D	ND	ND	H	ND	BoNT/D	Y
BD178F	AA	Y	D, CD	ND	ND	I	I	BoNT/D	Y
BD180F	Y	Y	D, CD	ND	ND	J	J	BoNT/D	Y
BD190F	W	Y	D, CD	ND	ND	-	-	BoNT/D	Y
BD174F	V	Y	C, DC, D, CD	ND	-	-	-	BoNT/D	Y
BD192F	V	Y	C, DC, D, CD	ND	-	-	-	BoNT/D	Y

^a^ Underlined VHHs were selected for generating VHH multimers. ^b^ Based on ELISA data of Figure 1 using an absorbance value of 0.5 as cutoff for binding. Binding to H_C_ domain is indicated as yes (Y) when any ELISA shows H_C_ binding. Similarly, binding to a particular BoNT serotype is based on binding in any of the ELISAs done with this serotype. ^c^ Based on DAS ELISAs presented in Figure 2. Dashes indicate that the antigenic site could not be determined. ^d^ ND, not determined. ^e^ No binding to BoNT/DCi in DAS ELISA (Figure 2) or Octet Red96 (Figure S3G). ^f^ No binding in ELISA to BoNT/Ci holotoxin.

**Table 3:** Affinities of VHHs for BoNT/Ci and DCi determined by biolayer interferometry.

				Multimer				Yeast VHH	
	BoNT/Ci			BoNT/Ci	BoNT/DCi			Production	Selection
	*K_D_* ^a^	*k_a_* × 10^5^	*k_d_* × 10^−5^	Affinity	*K_D_* ^a^	*k_a_* × 10^5^	*k_d_* × 10^−5^	Level	for BoNT
VHH	(pM)	(1/Ms)	(1/s)	Increase ^b^	(pM)	(1/Ms)	(1/s)	(mg/L) ^c^	C/DC
BC24F	504	3.1	16		ND ^d^			6.2	
BC24BC123F	143	6.0	8.6	3.5	719	3.9	28	3.5	
BC24BD157F	ND				70	6.1	4.3	3.6	DC
BC24BD165F	ND				120	7.2	8.6	2.8	
BC123F	110	5.9	6.5		ND			12	
BC123BC134F	75	8.0	6.0	1.5	65	15	9.7	5.0	DC
BC123BC147F	<3	11	<0.28 ^e^	>43	93 ^f^	8.0	7.4	26	C
BC123BC24F	37	7.7	2.9	2.9	190	5.7	11	1.7	
BC123BC134BC126F	5	33	1.8	4.5	ND			2.1	
BC123BC134BC147F	<1	21	<0.28	>82	ND			8.6	
BC123BD157F	ND				107	15	16	5.1	
BC123BD165F	ND				145	12	17	1.2	
BC126F	25	4.8	1.2		ND			1.7	
BC126BC123F	7	12	0.86	3.3	ND			7.6	
BC126BC141F	12	12	1.4	2.0	ND			2.6	
BC126BC147F	<3	8.9	<0.28	>7.9	ND			15	C
BC126BC24F	10	13	1.3	2.5	ND			7.7	
BC126BC123BC147F	6	23	1.3	4	508	5.6	29	8.4	C
BC126BC123BC24F	19	19	3.5	1	111	7.0	7.7	3.7	C
BC134F	166	8.2	14		ND			57	
BC134BC123F	28	11	3.1	3.9	89	8.3	7.4	8.4	
BC134BC147F	<6	4.6	<0.28	>28	386 ^g^	15	58	99	C
BC141F	482	2.9	14		ND			1.4	
BC141BC123F	<5	5.9	<0.28	>23	279	7.4	21	2.8	C
BC141BC126F	21	7.7	1.6	1.2	ND			2.1	
BC141BC147F	77	4.1	3.2	6.3	ND			1.9	
BC141BC24F	20	5.7	1.2	24	285	3.2	9.1	11	
BC147F	2520	0.4	10		ND			13	
BC147BC126F	69	6.5	4.5	0.4	ND			18	
BC147BC134F	131	5.0	6.6	1.3	ND			46	
BC147BC141F	201	2.6	5.3	2.4	ND			29	
BC147BC24F	<12	2.4	<0.28	>43	236	2.7	6.5	50	C
BD157BC24F	ND				155	7.7	12	3.1	
BD157BC123F	ND				148	9.2	14	6.5	
BD157BC123BC24F	ND				154	7.0	11	2.1	
BD165BC24F	ND				99	5.7	5.6	2.0	DC
BD165BC123F	ND				130	9.8	13	1.4	
BD165BC123BC24F	ND				265	6.0	16	2.5	

^a^ Different *K_D_* values are visualized by red/blue coloring. ^b^ Increase in affinity is the *K_D_* ratio of monovalent corresponding VHH with highest affinity versus multimeric VHH. ^c^ Yeast VHH production level is based on the VHH yield after IMAC purification from a single 0.5 L culture. ^d^ ND, not determined. ^e^ A *k_d_* < 0.28 × 10^−5^ 1/s indicates the detection limit of the assay. ^f^ Multimers with underlined *K_D_* values are expected to bind monovalently to BoNT/DCi, based on reactivity to soluble BoNT/DCi. ^g^ R^2^ is 0.96, indicating bad curve fitting to a 1:1 interaction model (see also Figure S3F).

**Table 4:** Affinities of VHHs for BoNT/Di and CDi determined by biolayer interferometry.

				Multimer				Yeast VHH	
	BoNT/Di			BoNT/Di	BoNT/CDi			Production	Selection
	*K_D_* ^a^	*k_a_* × 10^5^	*k_d_* × 10^−5^	Affinity	*K_D_* ^a^	*k_a_* × 10^5^	*k_d_* × 10^−5^	Level	for BoNT
VHH	(pM)	(1/Ms)	(1/s)	Increase ^b^	(pM)	(1/Ms)	(1/s)	(mg/L) ^c^	D/CD
BD157F	85	5.6	4.8		ND ^d^			20	
BD157BD166F	117	5.4	6.3	0.5	ND			17	
BD165F	192	7.0	13		ND			2.2	
BD165BD166F	63	6.2	3.9	1.0	ND			2.8	
BD166F	62	3.1	1.9		ND			1	
BD166BD157F	<5	5.8	<0.28 ^e^	>13	ND			15	D
BD166BD165F	20	5.1	1.0	3.1	ND			5.9	
BD166BD172F	79	4.1	3.3	0.8	60	3.8	2.3	2.3	D, CD
BD166BD178F	229	5.0	12	0.3	180	3.9	7.0	16	
BD166BD180BD172F	128	4.6	5.9	0.5	180	3.5	6.3	2	
BD166BD180BD178F	115	4.7	5.4	0.5	122	4.2	5.1	8.9	
BD166BD180F	496	1.1	5.4	0.1	103	4.2	4.3	22	
BD172F	711	3.6	26		ND			3.0	
BD172BD166F	39	6.9	2.7	1.6	57	8.3	4.7	1.5	D, CD
BD172BD178F	156	6.7	11	2.1	227	7.9	18	2.7	
BD172BD180F	298	4.6	14	2.4	739	7.1	52	1.4	
BD178F	327	9.6	31		ND			17	
BD178BD166F	64	7.5	4.8	1.0	68	9.2	6.2	17	D, CD
BD178BD172F	88	6.3	5.6	3.7	85	8.3	7.0	7.2	
BD178BD180F	116	5.4	6.2	2.8	208	8.1	17	22	
BD180F	1520	2.9	44		ND			14	
BD180BD166F	73	4.1	3.0	0.9	96	5.6	5.3	14	
BD180BD172F	50	2.2	1.1	14	238	3.0	7.2	2.2	
BD180BD178F	135	3.3	4.5	2.4	281	5.2	15	14	
BD180BD166BD172F	124	6.3	7.8	0.5	59	7.3	4.3	0.9	
BD180BD166BD178F	14	3.9	0.54	4.6	99	5.6	5.5	6.3	D, CD

^a^ Different *K_D_* values are visualized by red/blue coloring. ^b^ Increase in affinity is *K_D_* ratio of corresponding monovalent VHH with highest affinity versus multimeric VHH. ^c^ Yeast VHH production level is based on the VHH yield after IMAC purification from a single 0.5 L culture. ^d^ ND, not determined. ^e^ A *k_d_* < 0.28 × 10^−5^ 1/s indicates the detection limit of the assay.

**Table 5:** Recombinant BoNTs used in this study.

BoNT Protein	BoNT Protein, Full Name ^a^	*C. botulinum* Strain	Protein ID	Amino Acid Residues	Tags	Reference
BoNT/Ai	BoNT/A	62A	AAA23262	-	His6, Strep	[65]
BoNT/A	scH6tBoNTAiS	62A	AAA23262	-	None	[65]
BoNT/Bi	scBoNTBiSL	Okra	AB232927	-	Strep	[65]
BoNT/B	BoNT/B	Okra	AB232927	-	None	[65]
BoNT/Ci	scH6tBoNTCiSL	468C phage C-St	CAA37780	-	His6, Strep	[16]
BoNT/C	BoNT/C	468C phage C-St	CAA37780	-	None	[65]
BoNT/DCi	scBoNTDCi	OFD05	BAH84873	-	None	[64]
BoNT/Di	scH6tBoNTDiSL	BVD/-3	CAA38175	-	His6, Strep	[16]
BoNT/D	BoNT/D	BVD/-3	CAA38175	-	None	[65]
BoNT/Cdi	scBoNTCDitSH	003-9	BAD90568	-	His6, Strep	[16]
BoNT/Ei	scBoNTEiS	Beluga	CAA43999	-	Strep	-
BoNT/Fi	scBoNTFitSH	Langeland	CAA57358	-	His6, Strep	-
H_C_/C	H_C_CS	468C phage C-St	CAA37780	867-1291	Strep	[16]
H_C_/DC	H_C_DCS	OFD05	BAH84873	863-1285	Strep	[64]
H_C_/D	H_C_DS	BVD/-3	CAA38175	863-1276	Strep	[16]
H_C_/CD	H_C_CDS	003-9	BAD90568	867-1280	Strep	-
LC/C	LC/C6xHN	468C phage C-St	CAA37780	1-436	6xHN	-
LC/D	LC/D6xHN	BVD/-3	CAA38175	1-436	6xHN	-

^a^ Product name used by Toxogen GmbH.

**Table 6:** Native neurotoxin complexes produced by *C. botulinum* cultures.

BoNT	*C. botulinum*	Toxin Concentration in DAS ELISA (µg/mL)	
Type	Strain	BoNT/C ELISA	BoNT/D ELISA
C	CKIII	0.82	ND ^a^
DC	16564	ND	ND
D	1873	ND	1.2
CD	2286-3	ND	ND

^a^ ND, not determined.

## Data Availability

Only CDR3 sequences of the isolated VHHs are disclosed. VHH proteins can be obtained from the authors at prizes covering their production costs. All other data are included in the manuscript.

## Supplementary Materials

The following supporting information can be downloaded at: https://www.mdpi.com/article/10.3390/toxins15090573/s1, Figure S1: Reducing SDS-PAGE analysis of yeast-produced VHH monomers and multimers; Figure S2: Construction of 52 VHH multimers; Figure S3: Analysis of VHH binding to inactivated recombinant BoNT by biolayer interferometry using an Octet Red96 biosensor; Figure S4: Effect of 2 M NaCl on VHH binding to inactivated recombinant BoNT analyzed by biolayer interferometry using an Octet Red96 biosensor.
