# Supplementary material for: Single-Domain Antibody Multimers for Detection of Botulinum Neurotoxin Serotypes C, D, and Their Mosaics in Endopep-MS
Source: Toxins (Basel). 2023 Sep 17;15(9):573. doi: 10.3390/toxins15090573 (PMC10535107; doi:10.3390/toxins15090573)
Supplement: Supplementary file 1 [file toxins-15-00573-s001.zip › toxins-2596340-supplementary.pdf]

Supplementary Materials: Single-Domain Antibody Multimers for Detection of Botulinum Neurotoxin Serotypes C, D, and Their Mosaics in Endopep-MS

Michiel M. Harmsen, Jan C. Cornelissen, Fimme J. van der Wal, Jan H. W. Bergervoet and Miriam Koene

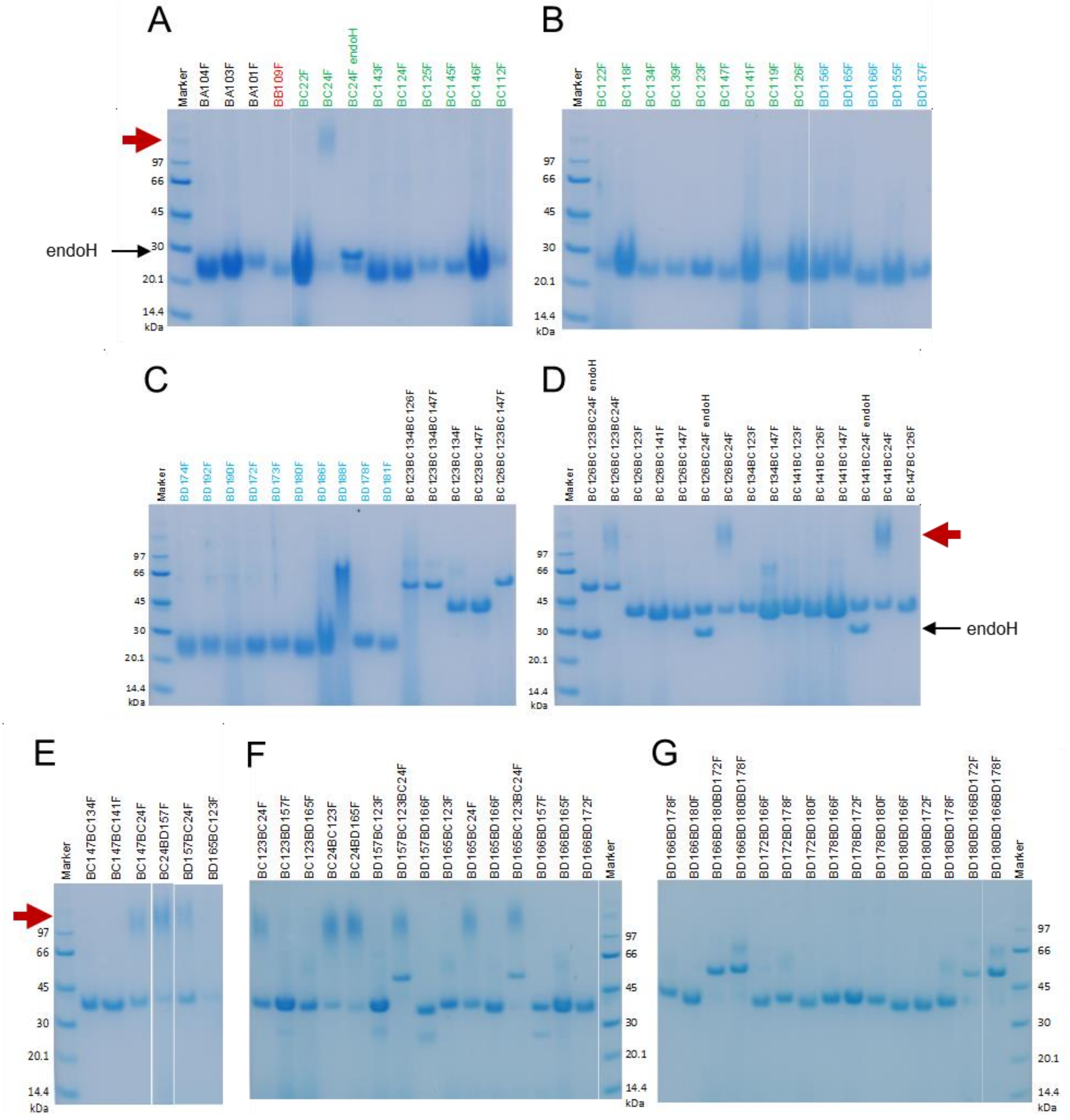

Figure S1. Reducing SDS-PAGE analysis of yeast-produced VHH monomers and multimers. Each panel represents an individual gel. Some VHHs were deglycosylated by treatment with endoglycosidase H prior to SDS-PAGE. In the corresponding lanes the position of the endoglycosidase H enzyme (endoH) is indicated (30 kDa; black arrow). N-glycosylated BC24F and N-glycosylated multimers containing BC24 are also indicated (big red arrow). The molecular weight of individual marker proteins is indicated next to each gel.

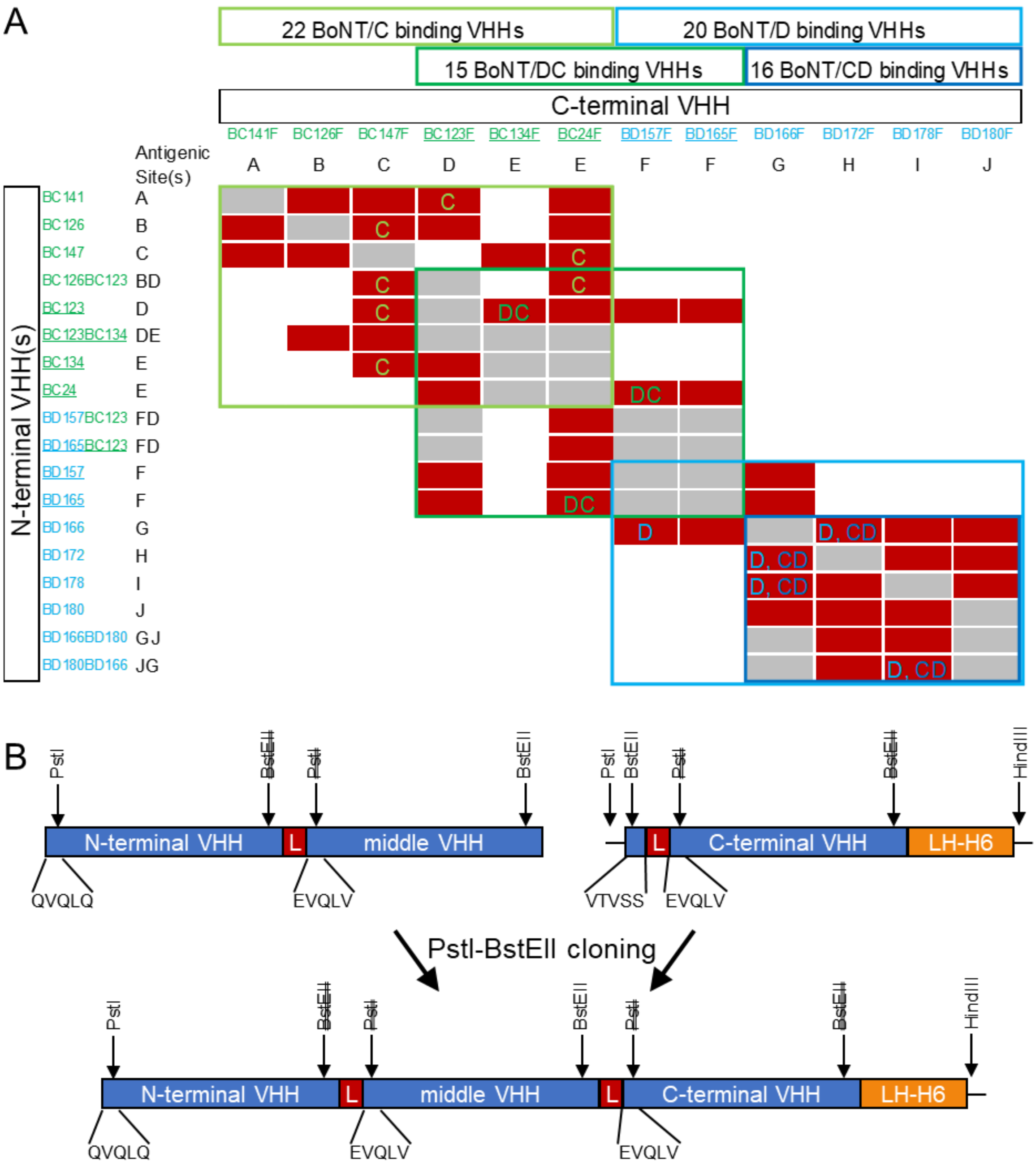

Figure S2. Construction of 52 VHH multimers. (**A**) Matrix of combinations of N-terminal VHH monomers or dimers with C-terminal VHHs. In total 52 multimers were produced (red boxes). Multimers with increased affinity selected for further evaluation in Endopep-MS are indicated with coloured letters C, D, DC and CD. Underlined VHHs bind to BoNT/DC in DAS ELISA. VHH multimers are named according to their constituent VHH monomers from N- to C-terminus. C-terminal and N-terminal VHHs were arranged according to their antigenic site(s) recognized. Blue and green boxing indicate the multimeric binding of VHHs to the 4 BoNT types as indicated on top. Multimeric BoNT/DC binding of BC123BC134BC126F and BC123BC134BC147F was ignored since BC123BC134F was also produced. Combinations of N- and C-terminal VHHs recognizing the same antigenic site were not selected for multimer generation (grey). (**B**) Cloning scheme of VHH trimers. Several constructs with C-terminal VHHs lacking internal PstI and BstEII sites were used for insertion of suitable VHH monomers or dimers through PstI-BstEII cloning. The resulting VHH trimers contained an N-terminal VHH starting with residues QVQLQ as in monomeric VHHs while middle and C-terminal VHHs started with EVQLV. VHH domains were linked using a (GGGGS)3 linker (L) and contained a C-terminal long hinge and hexahistidine tag (LH-H6). Destroyed restriction sites are indicated by double strikethrough.

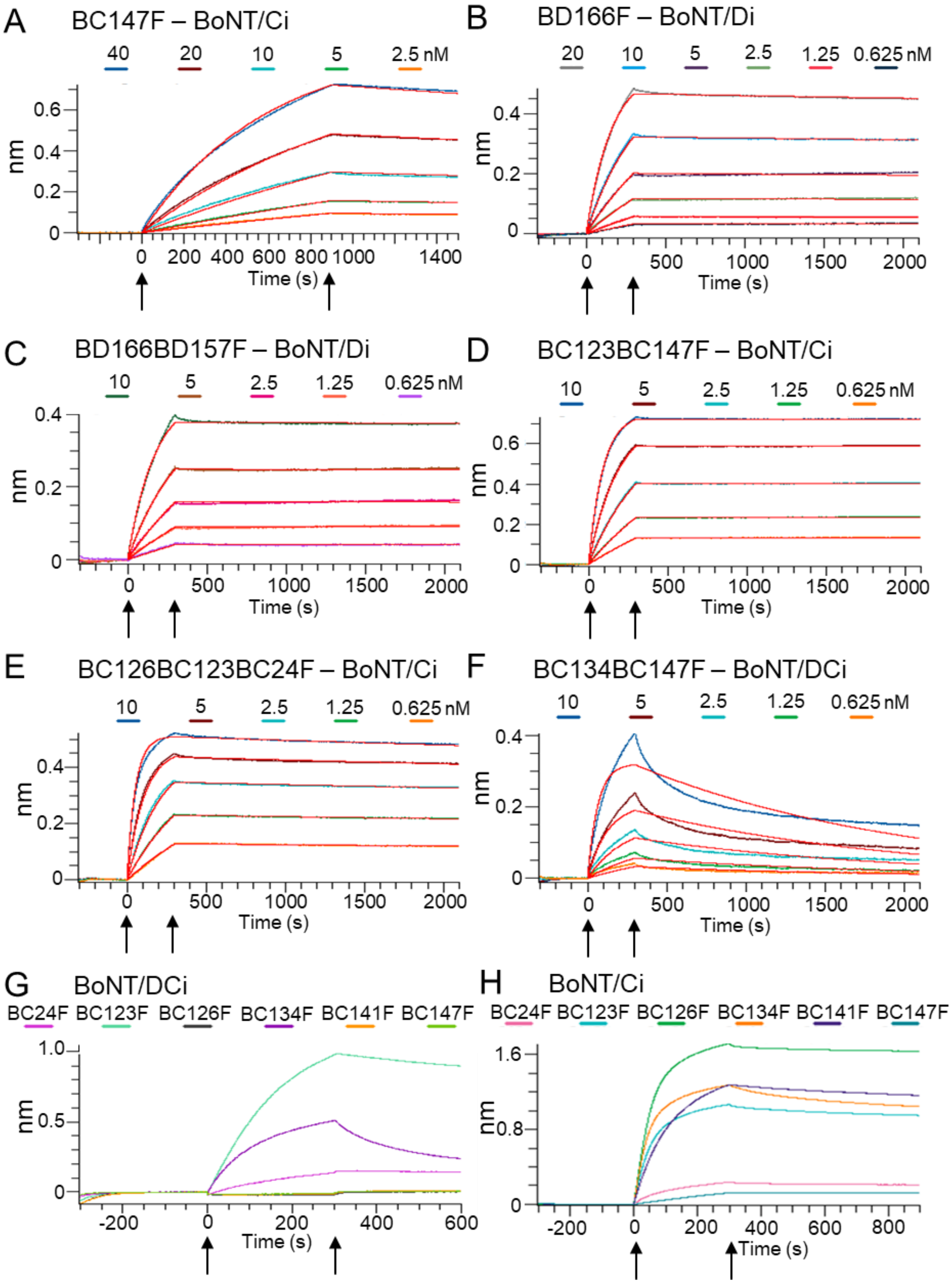

Figure S3. Analysis of VHH binding to inactivated recombinant BoNT by biolayer interferometry using an Octet Red96 biosensor. Biotinylated VHHs were coupled to SAX sensors. The graphs show the three incubations of VHH loaded sensors, demarcated by arrows, in buffer until t=0 (baseline), association phase with BoNT protein and dissociation phase in buffer. (**A-F)** Affinity determination of individual VHHs binding to different BoNT proteins, as indicated on top. Each panel depicts data for one mono- or multimeric VHH interacting with a specific BoNT protein at different concentrations that were used for global curve fitting to a 1:1 interaction model. Red lines are the fitted curves. BC134BC147F binding to BoNT/DCi (**F**) resulted in a relatively bad curve fit (R^2 = 0.96) while all other affinity measurements resulted in good curve fitting (R^2 > 0.98). (**G,H**) Scouting experiment with 30 nM BoNT/DCi (**G**) or BoNT/Ci (**H**) of sensors loaded with 6 biotinylated VHH monomers (1 µg/ml) that were also used for construction of multimers. BC126F, BC141F and BC147F do not bind BoNT/DCi.

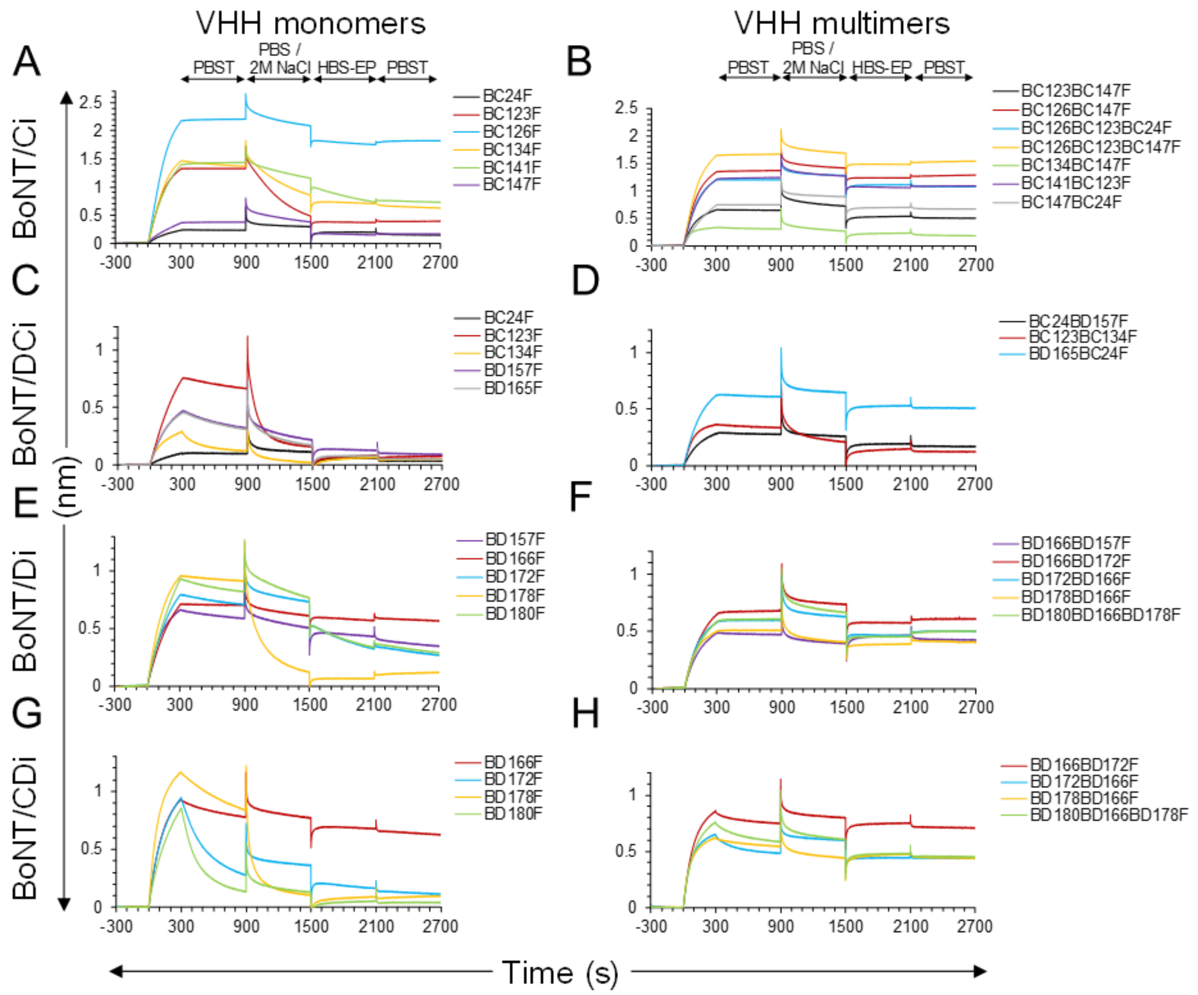

Figure S4. Effect of 2 M NaCl on inactivated recombinant BoNT dissociation from VHHs analysed by biolayer interferometry using an Octet Red96 biosensor. Biotinylated VHH monomers (**A,C,E,G**) or multimers (**B,D,F,H**) were loaded on SAX biosensors and a baseline in PBST was done from -300 to 0 sec. Then 20 nM BoNT/Ci (**A,B**), BoNT/DCi (**C,D**) BoNT/Di (**E,F**) or BoNT/CDi (**G,H**) analyte was captured. BoNT dissociation was subsequently measured during 4 subsequent incubations of 600 sec in PBST, PBS / 2 M NaCl, HBS-EP and PBST. The sensorgrams show the baseline in PBST for 300 sec obtained after VHH loading, the association of BoNT from 0 to 300 sec, followed by four dissociation phases of 600 sec in four buffers.
